# A Novel Pathogenic Variant in *PAX2*‐Related Renal Coloboma Syndrome Identified by Prenatal Diagnosis: A Case Report and Literature Review

**DOI:** 10.1155/crog/5112963

**Published:** 2026-07-06

**Authors:** Gang Wang, Jie Wang, Lichun Zhang, Dongxia Hou, Yan Huang, Hong Wang, Xueyuan Zhou, Xiaoyan Pang, Lu Li, Yuexin Song, Xiaohua Wang

**Affiliations:** ^1^ Department of Genetics, Inner Mongolia Maternity and Child Health Care Hospital, Hohhot, Inner Mongolia Autonomous Region, China; ^2^ Inner Mongolia Engineering Research Center of Medical Genetics, Hohhot, China; ^3^ Department of Obstetrics and Gynecology, Inner Mongolia Maternity and Child Health Care Hospital, Hohhot, Inner Mongolia Autonomous Region, China

**Keywords:** genetic disease, *PAX2*, prenatal diagnosis, renal coloboma syndrome, whole-exome sequencing

## Abstract

**Introduction:**

Renal coloboma syndrome (RCS) is an autosomal dominant disorder caused by pathogenic variants in the *PAX2* gene, primarily affecting renal and optic nerve development. However, the presentation of RCS is highly heterogeneous, ranging from mild renal anomalies to severe multi‐organ involvement. This phenotypic variability often poses significant challenges for accurate clinical diagnosis.

**Case Presentation:**

In this study, a novel heterozygous *PAX2* missense mutation (NM_000278.5: c.404 T > G, p.Ile135Ser) was identified via whole‐exome sequencing (WES) in a 31‐year‐old pregnant woman and her fetus. According to the American College of Medical Genetics and Genomics (ACMG) guidelines, this mutation is classified as likely pathogenic. The phenotypic divergence observed between the mother and neonate underscores the syndrome′s variable penetrance. While the mother exhibited relatively mild renal and optic nerve anomalies, the neonate presented with severe multi‐organ involvement, including renal structural defects, hearing impairment, and extensive pulmonary, cardiac, and cerebral lesions, culminating in fatal intracranial hemorrhage and multi‐organ failure at 2 months of age.

**Discussion:**

The present case identified a previously unreported pathogenic variant in the *PAX2* gene, thereby expanding the mutational spectrum of *PAX2*‐related RCS. It also further underscores the phenotypic heterogeneity of this disorder, even among members of the same family. Additionally, the genotype–phenotype spectrum of *PAX2*‐related cases was also reviewed to facilitate early diagnosis, management, and genetic counseling for RCS.

## 1. Introduction

Human paired box gene 2 (*PAX2*), located at 10q24.31, spans approximately 84 kb and comprises 12 exons. It is indispensable for regulating transcription factors that orchestrate the development of the urogenital tract, central nervous system, eyes, and ears. Moreover, *PAX2* plays a pivotal role in the differentiation and proliferation of renal cells [1]. During early fetal development, *PAX2* expression is essential for maintaining the progenitor cell population within the nephrogenic zone, thereby ensuring proper nephron formation [2]. Both overexpression and underexpression of *PAX2* can disrupt growth and development. Pathogenic mutations in *PAX2* have been firmly linked to congenital anomalies of the kidney and urinary tract (CAKUT). The associated disorder, known as renal coloboma syndrome (RCS) or papillorenal syndrome, follows an autosomal dominant inheritance pattern [3]. Affected individuals may progress to end‐stage kidney disease, necessitating dialysis or transplantation. The syndrome typically presents with optic nerve defects and renal dysplasia, manifesting as focal segmental glomerulosclerosis (FSGS), multicystic dysplastic kidneys, uric acid nephrolithiasis, vesicoureteral reflux, high‐frequency hearing loss, retinal coloboma, and visual impairment [4]. Notably, *PAX2*‐related diseases exhibit marked clinical variability and phenotypic heterogeneity, even among members of the same family [5, 6]. With the increasing integration of genetic diagnostics into clinical practice, a growing number of *PAX2* variant‐associated cases have been identified. Reported mutation types encompass base substitutions, deletions, insertions, and duplications. All of these pathogenic alterations can precipitate dysplasia and abnormalities across various tissues and organs [7].

In this study, a novel heterozygous *PAX2* mutation, classified as likely pathogenic, was identified through whole‐exome sequencing (WES) in prenatal diagnosis. Both the pregnant woman and the newborn carrying the variant exhibited RSC‐related phenotypes, but there were notable differences in the affected organs and disease severity. Additionally, the genotype–phenotype spectrum of cases with *PAX2* variants was reviewed, aiming to provide guidance and reference for the prenatal diagnosis and early intervention of affected fetuses and newborns.

## 2. Case Presentation

### 2.1. Prenatal Examination

A 31‐year‐old pregnant woman was admitted to the Inner Mongolia Maternity and Child Health Care Hospital for a routine antenatal examination at 18 weeks of gestation. The ultrasound indicated fluid accumulation in the fetal abdominal cavity and enhanced intestinal echo. Subsequently, an amniocentesis test was performed. No abnormality was found in the fetal karyotype (46, XN), and the chromosomal microarray analysis (CMA) showed a negative result. At 23 weeks gestation, the pregnant woman presented a single live fetus in the uterus with severe oligohydramnios. No obvious renal echo was detected in the fetus, and bilateral kidneys were not visible. Four days later, a repeat ultrasound and magnetic resonance imaging (MRI) detected a small volume of both kidneys. The structure of the right kidney was atypical, which was suspected to encompass a double renal pelvis accompanied by pyelocaliectasis. Mild hydronephrosis was observed in the left kidney. Bilateral ureters were not dilated. The bladder was well filled, and the wall was thin and smooth featuring ideal sound transmission. Cystic signals appeared in both lungs of the fetus which were irregular in shape with multiple pulmonary septa.

**Figure 1:**
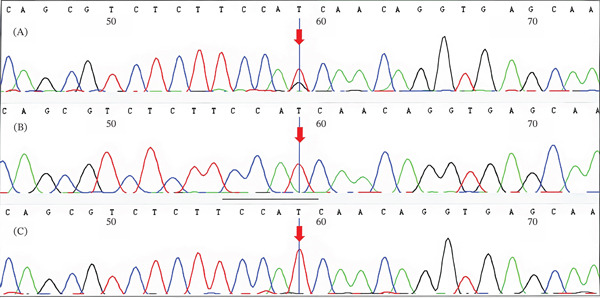
Validation of *PAX2* mutation in the (A) fetus and (B and C) his grandparents. The arrow indicates the location of the mutation.

**Figure 2:**
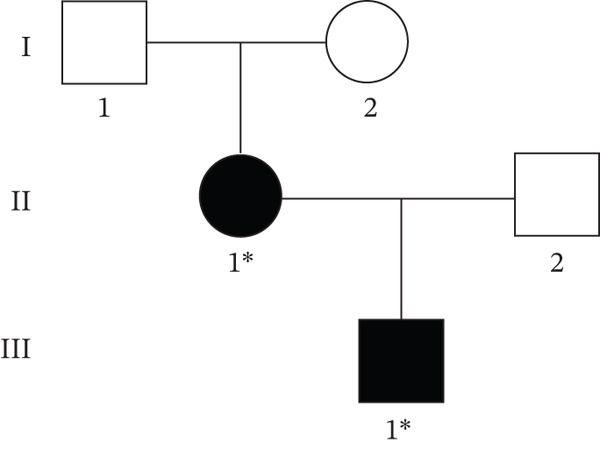
Pedigree of the family in this study. II‐1, the pregnant woman carrying a *PAX2* variant; II‐2, the husband of the pregnant woman; I‐1 and I‐2, the parents of the pregnant woman; III‐1, the newborn carrying a *PAX2* variant.

To investigate whether the observed fetal anomalies were linked to genetic alterations, trio whole‐exome sequencing (trio‐WES) was conducted for the family using the MGISEQ‐2000 platform (MGI Tech Co. Ltd., China), following informed consent. The analysis identified a previously unreported heterozygous mutation, NM_000278.5: c.404 T > G (p.Ile135Ser), located in Exon 3 of the *PAX2* gene in both the pregnant woman and the fetus. This nucleotide substitution (T > G) leads to an isoleucine‐to‐serine change at the protein level. No record about this variant was retrieved in gnomAD database, and Sanger sequencing confirmed its absence in the pregnant woman′s parents (Figure 1). It was indicated that the missense mutation represents a de novo event in the pregnant woman, subsequently transmitted to the fetus (Figure 2). This variant was predicted to be deleterious by multiple computational tools, with a REVEL score of 0.97. The pregnant woman reported a prior diagnosis of optic nerve atrophy, a right renal duplex system, and mild hydronephrosis in the left kidney—symptoms that align with the clinical spectrum of *PAX2*‐related disorders. According to the American College of Medical Genetics and Genomics (ACMG) guidelines, the *PAX2* variant (NM_000278.5: c.404 T > G, p.Ile135Ser) was classified as likely pathogenic based on the currently available evidence.

At 35 weeks of gestation, oligohydramnios persisted on antenatal ultrasound. The fetus demonstrated intrauterine growth restriction and exhibited bilaterally small kidneys with increased echogenicity, consistent with polycystic renal dysplasia.

### 2.2. Neonatal Examination and Follow‐Up

At 37 weeks and 3 days of gestation, a male newborn was delivered via spontaneous vaginal delivery, with a birth weight of 1.84 kg. The infant presented with bipedal valgus. Radiographic examination revealed exaggerated and indistinct pulmonary markings, accompanied by a slight increase in pulmonary field transparency and excessive intestinal gas shadows. Ultrasonography indicated that the kidneys were small in size, with enhanced parenchymal echogenicity, multiple anechoic lesions, and an ill‐defined boundary between the cortex and medulla. The right scrotum was empty, and no testicular echo was detected in the right groin. Cardiac assessment identified a patent ductus arteriosus and a patent foramen ovale, along with slight thickening of the left ventricular wall and a mild tricuspid regurgitation signal observed during systole. Serological tests showed elevated levels of N‐terminal pro‐B‐type natriuretic peptide (NT‐proBNP), creatinine, uric acid, and blood urea nitrogen, as well as hypocalcemia. Brain MRI revealed bilateral temporal lobe hematomas with subdural hemorrhage and a small amount of effusion in the middle ear mastoid. No obvious structural abnormalities were detected in both eyes, but the infant exhibited hearing impairment in the left ear.

Despite being thoroughly informed of the infant′s unstable condition and high risk, the parents were discharged 1 week after birth. In subsequent follow‐up, we learned that the baby died at 2 months of age due to an intracranial hemorrhage and multi‐organ failure.

## 3. Discussion

Patients harboring homozygous *PAX2* mutations typically succumb shortly after birth due to severe organ loss or damage. In contrast, individuals with heterozygous variants exhibit a wide spectrum of disease manifestations. RCS is a classic phenotype of *PAX2*‐related disorders. *PAX2* plays a crucial role in the interaction between the ureteric bud and metanephric mesenchyme during podocyte formation, a key component of the glomerular filtration barrier. Podocyte dysfunction is a primary driver of proteinuria and progressive renal injury [8]. Pathogenic variants can disrupt the delicate balance between cell proliferation and apoptosis within renal tissue, leading to aberrant renal development and function. Additionally, dysregulated *PAX2* expression can impede the differentiation of nephron progenitor cells. Overexpression of *PAX2* promotes the fusion of the ureteric bud with the metanephric mesenchyme, resulting in polycystic renal dysplasia and ureteral atresia, whereas insufficient expression leads to renal hypoplasia and ureteral malformations [9]. Among those with pathogenic *PAX2* variants, over 92% present with renal structural or functional abnormalities, 77% exhibit visual defects, and 7% experience hearing impairment, underscoring the extensive phenotypic heterogeneity associated with *PAX2*‐related diseases [10]. Clinical presentations can vary dramatically, even among family members carrying the identical variant (Table 1). Some individuals suffer multi‐organ involvement, including renal, ocular, and auditory systems, whereas others display isolated renal or optic nerve anomalies [6]. The severity of renal malformations also spans a broad spectrum, ranging from asymptomatic cases to severe renal failure. For instance, Patient 7, who possessed a frameshift mutation (c.76dupG, p.Val26Glyfs∗28) in *PAX2*, required dialysis on the 13th day after birth due to rapidly deteriorating renal function and subsequently succumbed to septic shock and multi‐organ failure at 9 months of age. In contrast, her father (Patient 8), who carried the same variant, experienced a slower progression to chronic kidney disease Stage 3, indicating a considerably milder disease course. In the current study, the *PAX2* variant (c.404 T > G, p.Ile135Ser) was identified concurrently in a woman and her fetus, yet they displayed markedly different clinical manifestations and disease severities. The pregnant woman exhibited abnormalities in both the kidneys and optic nerve, whereas the newborn presented with renal dysplasia, hearing impairment, and multiple injuries affecting the lungs, heart, and brain, but without any visual loss. Unfortunately, the newborn died 2 months after birth, precluding any further follow‐up on disease progression. Although cases of *PAX2* mutations continue to be reported, the factors influencing disease progression remain largely unclear. The age of onset and the timeline to renal failure vary significantly among individuals [10]. Consequently, long‐term follow‐up and regular examinations are essential for carriers of *PAX2* mutations. At present, there is still a lack of specific treatments for RCS. A definitive diagnosis of this highly heterogeneous disease through genetic analysis can help avoid the unnecessary use of immunosuppressive agents, which often have significant side effects, and facilitate early treatment and intervention for individuals harboring pathogenic mutations.

**Table 1:** Genotype and phenotype of reported cases with *PAX2* mutation.

No.	Age (y)	Sex	Mutation	Type	Renal manifestation	Visual deficit	Hearing loss	Reference
1	8	M	c.959C > G (p.Ser320∗)	Nonsense	Proteinuria; FSGS	No	No	[11]
2	36	M	c.76delG (p.Val26Cysfs∗3)	Frameshift	FSGS	Yes	NR	[12]
3	46	F	c.175C > T (p.Arg59Trp)	Missense	No renal disorders	Yes	NR	[13]
4	1.3	F	c.94C > T (p.Pro32Ser)	Missense	Renal cysts	No	No	[14]
5	36	F	c.219C > G (p.Tyr73∗)	Nonsense	Early‐onset ESRD	Yes	NR	[15]
6	19	M	c.70dupG (p.Leu23fs)	Frameshift	ESRD	Yes	Yes	[16]
7	0^a^	F	c.76dupG (p.Val26Glyfs∗28)	Frameshift	ESRD	Yes	Yes	[16]
8	34	M	c.76dupG (p.Val26Glyfs∗28)	Frameshift	CKD Stage III	Yes	NR	[16]
9	6	M	c.70_72delinsA (p.Gly24Argfs∗29)	Truncation	FSGS progressed to ESRD	Yes	NR	[17]
10	11	M	c.350G > C (p.Arg117Pro)	Missense	CKD Stage IV	Yes	NR	[18]
11	23	F	c.430C > T (p.Gln144Ter)	Nonsense	Oligomeganephronia	No	No	[19]
12	37	M	c.130C > G (p.Leu44Val)	Missense	Adult‐onset ESRD	No	NR	[20]
13	13	F	c.213‐2A > G (intron 2)	Splicing	Renal function alteration	Yes	NR	[21]
14	26	M	c.389C > G (p.Pro130Arg)	Missense	Renal failure	Yes	NR	[21]
15	15	M	c.988G > A (p.Gly330Ser)	Missense	No renal disorders	Yes	NR	[21]

*Note:* Age is expressed in years (y).

Abbreviations: CKD, chronic kidney disease; ESRD, end‐stage renal disease; F, female; FSGS, focal segmental glomerulosclerosis; M, male; NR, not reported.

^a^For individuals younger than 1 year, the age is shown as “0 y”.

The ACMG′s guidelines for interpreting sequence variants categorize genetic alterations into five classes, including pathogenic (P), likely pathogenic (LP), variant of uncertain significance (VUS), likely benign (LB), and benign (B). Among these, P/LP and B/LB indicate a > 90% certainty that a variant is disease‐causing or benign, respectively. In contrast, VUS denotes a temporary uncertainty regarding the association between a gene mutation and disease, pending further evidence [22]. With additional supporting data, a VUS can be reclassified as either P/LP or B/LB. The interpretation of sequence variants remains a significant challenge due to conflicting evidence and insufficient scientific data regarding their functional properties and clinical relevance. Misclassifying or overlooking potentially pathogenic genetic alterations can prevent patients from receiving accurate diagnoses and appropriate treatments, thereby adversely affecting genetic counseling and disease management [23, 24]. In this study, the newly identified missense mutation c.404 T > G (p.Ile135Ser) in the *PAX2* gene was evaluated as LP based on the following evidence: (1) PM6 (de novo): This variant was identified as a de novo mutation in the proband. However, the parentage verification was performed solely using Sanger sequencing within the family and lacked rigorous confirmation of the biological relationship between the parents and the proband. (2) PM2_Supporting (absent from controls): No records of this variant were found in public population databases. (3) PP3_Strong (computational evidence): Multiple in silico prediction tools consistently indicated that this variant would have a deleterious impact with a REVEL value of 0.97 [25].

WES is a targeted next‐generation sequencing (NGS) technique that focuses on sequencing the protein‐coding regions of human genomes, known as exons, which constitute approximately 1%–2% of the total DNA. It can be used to detect disease‐associated mutations in the exon regions of over 20,000 genes through massively parallel sequencing [26]. Compared to whole‐genome sequencing (WGS), the scope of WES is more concentrated, which aids in enhancing sequencing depth and significantly improves the detection rate of low‐frequency mutations and rare variants. It is more cost‐effective and efficient [27]. The higher resolution and sensitivity relative to karyotyping and CMA enable the precise identification of subtle variations such as single nucleotide substitutions, insertions, and deletions [28]. The smaller output datasets also reduce the complexity and difficulty of bioinformatics analysis. Furthermore, WES is not only capable of detecting known gene sites but can also uncover new genetic mutations. The application of WES in antenatal examination undoubtedly contributes to the elevation of detection rates for abnormal fetuses, realizing the accurate recognition of pathogenic variants, thereby facilitating early diagnosis, treatment, and genetic counseling. In this study, renal damage and developmental abnormalities were initially identified in a fetus through prenatal ultrasound examination. Subsequently, a previously unreported heterozygous variant (c.404 T > G, p.Ile135Ser) in *PAX2* was discovered by WES. Validation in family members revealed that the variant was a de novo mutation in the pregnant woman and was inherited by the fetus. The mutation site was located in the Exon 3 region of *PAX2*, which is responsible for encoding the N‐terminal DNA‐binding paired domain of the protein. The missense mutation causing an amino acid substitution may alter the protein structure and the stability of the paired domain, which affects the protein′s DNA‐binding function, potentially leading to developmental defects in kidneys and other organs [5]. The differences in symptoms and disease severity of the two patients further illustrate the clinical heterogeneity of *PAX2*‐related disorders. The discovery of the new mutation site also expands the genetic spectrum of RSC, providing guidance and reference for genetic testing.

## Author Contributions

Gang Wang: data curation, investigation, methodology, funding acquisition, and writing – original draft. Jie Wang: data curation, investigation, resources, and writing – review and editing. Lichun Zhang: resources, data curation, funding acquisition, and writing – review and editing. Dongxia Hou: resources, funding acquisition, and writing – review and editing. Yan Huang: data curation, resources, and writing – review and editing. Hong Wang: resources, investigation, and writing – review and editing. Xueyuan Zhou: resources, writing – review and editing. Xiaoyan Pang: resources, writing – review and editing. Lu Li: resources, writing – review and editing. Yuexin Song: resources, writing – review and editing. Xiaohua Wang: conceptualization, funding acquisition, project administration, supervision, and writing – review and editing. Gang Wang and Jie Wang have contributed equally to this work.

## Funding

This study was supported by the Science and Technology Project of the Public Hospital Research Joint Fund of Inner Mongolia Medical Academy, 2025GLLH0333; the Inner Mongolia Autonomous Region “Yingcai Xingmeng” Project‐Youth Elite Talent Training Program, Q202553; the Key Technology Research Plan Project of Inner Mongolia Autonomous Region, 2021GG0130; the Healthcare Science and Technology Plan Project of Inner Mongolia Health Commission, 202201138; and the Research Project of Inner Mongolia Maternity and Child Health Care Hospital, 2024FYYNB012.

## Disclosure

All authors have read and approved the final version of the manuscript.

## Ethics Statement

All investigations were performed in accordance with the ethical standards of the Institutional Committee and the World Medical Association Declaration of Helsinki. Written informed consent for publication of this case report was obtained from the participants, and they fully understood that their identity would remain confidential and no personally identifiable information would be published.

## Conflicts of Interest

The authors declare no conflicts of interest.

## Data Availability

The data that support the findings of this study are available from the corresponding author upon reasonable request.
